# Identifying which septic patients have increased mortality risk using severity scores: a cohort study

**DOI:** 10.1186/1471-2253-14-1

**Published:** 2014-01-02

**Authors:** Charis A Marwick, Bruce Guthrie, Jan EC Pringle, Shaun R McLeod, Josie MM Evans, Peter G Davey

**Affiliations:** 1 Population Health Sciences Division, Medical Research Institute, University of Dundee, Mackenzie Building, Kirsty Semple Way, Dundee DD2 4BF, UK; 2 School of Health and Life Sciences, K415 Buchanan House, Glasgow Caledonian University, Glasgow G4 0BA, UK; 3 Department of Anaesthesia, Ninewells Hospital & Medical School, Dundee DD1 9SY, UK; 4 University of Stirling, School of Nursing, Midwifery and Health, R G Bomont Building, Stirling FK9 4LA, UK

**Keywords:** Sepsis, Severity, Risk scores, Outcomes, Mortality, CURB, CURB65, Systemic inflammatory response syndrome, SIRS

## Abstract

**Background:**

Early aggressive therapy can reduce the mortality associated with severe sepsis but this relies on prompt recognition, which is hindered by variation among published severity criteria. Our aim was to test the performance of different severity scores in predicting mortality among a cohort of hospital inpatients with sepsis.

**Methods:**

We anonymously linked routine outcome data to a cohort of prospectively identified adult hospital inpatients with sepsis, and used logistic regression to identify associations between mortality and demographic variables, clinical factors including blood culture results, and six sets of severity criteria. We calculated performance characteristics, including area under receiver operating characteristic curves (AUROC), of each set of severity criteria in predicting mortality.

**Results:**

Overall mortality was 19.4% (124/640) at 30 days after sepsis onset. In adjusted analysis, older age (odds ratio 5.79 (95% CI 2.87-11.70) for ≥80y versus <60y), having been admitted as an emergency (OR 3.91 (1.31-11.70) versus electively), and longer inpatient stay prior to sepsis onset (OR 2.90 (1.41-5.94) for >21d versus <4d), were associated with increased 30 day mortality. Being in a surgical or orthopaedic, *versus* medical, ward was associated with lower mortality (OR 0.47 (0.27-0.81) and 0.26 (0.11-0.63), respectively). Blood culture results (positive *vs.* negative) were not significantly association with mortality. All severity scores predicted mortality but performance varied. The CURB65 community-acquired pneumonia severity score had the best performance characteristics (sensitivity 81%, specificity 52%, positive predictive value 29%, negative predictive value 92%, for 30 day mortality), including having the largest AUROC curve (0.72, 95% CI 0.67-0.77).

**Conclusions:**

The CURB65 pneumonia severity score outperformed five other severity scores in predicting risk of death among a cohort of hospital inpatients with sepsis. The utility of the CURB65 score for risk-stratifying patients with sepsis in clinical practice will depend on replicating these findings in a validation cohort including patients with sepsis on admission to hospital.

## Background

Sepsis carries a high risk of death, with estimated mortality rates for severe sepsis of up to 50% [1,2]. There is a substantial body of evidence that early initiation of aggressive therapy can reduce mortality [3,4], but this requires prompt recognition. Sepsis has been defined as the systemic inflammatory response syndrome (SIRS, Table 1) due to infection [5], with severe sepsis defined as sepsis with resulting organ hypoperfusion or dysfunction. Inconsistency exists across trials and guidelines as to the level of dysfunction, and of which organs, required to qualify as having severe sepsis [3,6-11]. This makes generalising research findings and developing local evidence-based clinical guidelines and protocols difficult. A simple and standardised way of identifying high risk sepsis patients is required to aid timely therapeutic decision-making.

**Table 1:** Tested severity criteria with the scoring system or severe sepsis definition from each

**Severity criteria**	**Scoring system/severe sepsis definition**
Systemic inflammatory response syndrome (SIRS) [5]	Two or more of (means SIRS): heart rate ≥90 beats/minute, respiratory rate ≥20 breaths/minute, temperature <36 or ≥38°C, white blood cell count <4 or ≥12 cells/mm^3^
Standardised early warning system (SEWS) score [12]	Sum of scores allocated for each clinical observation:* respiratory rate, oxygen saturation, temperature, SBP, heart rate, stimulus required to invoke a response
CURB65 pneumonia severity score [16]	One point for each of: new confusion, urea >7 mmol/l, respiratory rate ≥30 breaths/min, SBP <90 mmHg or DBP ≤60 mmHg, age ≥65 years
Institute for Healthcare Improvement website [8]	One or more of (means severe sepsis): SBP <90 mmHg or decreased by >40 mmHg, MAP <70 mmHg, lactate >2 mmol/l, SpO_2_ ≤90%, urine output <30 ml/hr, creatinine >177 mmol/l, INR >1.5, APTT >60secs, platelets <100/mm^3^, bilirubin >35 mmol/l
Surviving Sepsis Campaign guidelines 2008 [7]	One or more of (means severe sepsis): SBP <90 mmHg or decreased by >40 mmHg, MAP <70 mmHg, lactate >4 mmol/l, oliguria, altered mental status
Survive Sepsis™ website [13]	One or more of (means severe sepsis): SBP <90 mmHg, MAP <65 mmHg, lactate >2 mmol/l, SpO2 ≤90%, urine output <30 ml/hr, creatinine >177 mmol/l, INR >1.5, APTT >60secs, platelets <100/mm^3^, bilirubin >34 mmol/l

SBP = systolic blood pressure, DBP = diastolic blood pressure, MAP = mean arterial blood pressure, INR = international normalised ratio, APTT = activated partial thromboplastin time.

*Graded scores of 0–3 allocated for each observation, detailed in Additional file 1: Table S1.

Severity criteria designed to identify high risk patients are available, but it is unclear which criteria perform best in identifying people who have the highest mortality risk among those with sepsis. Potentially useful severity assessment tools for sepsis include generic tools such as the Standardised Early Warning System (SEWS) score [12], and sepsis-related criteria such as the number of SIRS criteria met [5] or the criteria for severe sepsis from the Institute for Healthcare Improvement (IHI) [8], the Surviving Sepsis Campaign (SSC) 2008 guidelines [7], or Survive Sepsis UK [13] (Table 1). A patient’s SEWS score (Table 1 and Additional file 1: Table S1) on admission to hospital correlated with in-hospital mortality and length of stay in a prospective audit [12], and meeting higher numbers of SIRS criteria has been associated with worse outcome in patients with bacteraemia [14] and with pneumonia [15]. The CURB65 severity score, which predicts mortality among patients with community-acquired pneumonia [16], may also apply to patients with sepsis. Despite being developed and validated in a different patient group, the criterion that make up the CURB65 score (Table 1) are each, with the exception of age, indicators of organ dysfunction, which in the context of sepsis means severe sepsis. The CURB65 score has been reported to correlate with mortality in emergency department patients with any infection [17], and we propose the score may also apply to hospital inpatients.

In this study, we evaluated the extent to which the above six sets of severity criteria and other clinical and demographic variables, were associated with 30 and 90 day mortality among a cohort of hospitalised patients who developed sepsis. We then compared the performance characteristics of each set of severity criteria in predicting mortality, and tested the potential to improve performance further by adding clinical variables to the best performing set of severity criteria.

## Methods

### Setting and data description

Ninewells Hospital is an 860-bedded, tertiary, teaching hospital serving the Scottish region of Tayside, which has a population of approximately 400,000. In this cohort study we retrospectively analysed data that had been collected prospectively. We identified patients who developed sepsis while inpatients in Ninewells Hospital in two cohorts, from September 2008 to February 2009 and October 2009 to March 2010, as part of a larger quality improvement project [18]. The study case definition was: an adult (≥18 years old) patient with sepsis occurring ≥24 h after admission to hospital, either as a first episode of sepsis or following a period of ≥24 h without meeting SIRS criteria. Patients with immune compromise due to chemotherapy or organ transplant were excluded. For the first cohort we screened patients who had blood cultures taken while in any hospital ward except paediatrics, obstetrics, haematology, oncology, acute admissions units, and the accident and emergency department, to identify patients meeting the study case definition. In pilot work, we demonstrated that screening patients who had blood cultures taken (regardless of whether positive or negative) had good performance characteristics for identifying patients with sepsis [18]. For the second cohort we restricted screening to general medical, general surgical, and orthopaedic wards (due to the design of the improvement project), which included 22 of the 30 wards screened for the first cohort.

The prospectively collected clinical data, which included the patient’s clinical measurements at sepsis onset, comorbidity, and demographics, were anonymised in a study database, with a Charlson Index of comorbidity [19] calculated for each patient. We linked these to routine data via the Health Informatics Centre (HIC), University of Dundee, using patients’ unique community health index (CHI) numbers. The Scottish Morbidity Record (SMR) databases accessed for this study were: SMR01 general acute inpatient and day case discharges, General Register Office for Scotland (GRO) death registrations, and the NHS Tayside Microbiology database. We obtained blood culture results and a Scottish Index of Multiple Deprivation (SIMD) quintile [20] for all patients and dates of all deaths up to 90 days after the end of data collection. Because the cohort included mainly older adults, we defined the age groups as <60 yrs, 60-69 yrs, 70-79 yrs and ≥80 yrs for analysis. We categorised blood culture results, from seven days before to seven days after sepsis onset, as positive, probable contaminant, or negative. The categorisation of isolated organisms was done by two Infectious Diseases physicians (CM and PD, authors) and a consultant microbiologist (GP, acknowledgements) (see Additional file 2: Table S2 for how we classified the isolated organisms). Patients were grouped according to their “most positive” blood culture result, so a patient with a positive culture and a probable contaminant was grouped as positive, and a patient with a probable contaminant and a negative culture was grouped as probable contaminant. In classifying sepsis severity, when the data item required for a severity criterion was not available we classified the patient as not meeting that criterion. This risked underestimating severity but reflected real-life performance of the criteria. For all definitions of severe sepsis, organ dysfunction must be new and distant from the site of infection, so we took comorbidity, previous blood results, and the site of infection into account when classifying severity criteria.

### Data analysis

We calculated 30 and 90 day mortality and used binary logistic regression to examine associations with age, gender, SIMD quintile [20], Charlson Index of comorbidity [19], admission type, ward type at sepsis onset, length of inpatient stay prior to sepsis onset, blood culture result, and by sepsis severity according to each set of criteria, with results reported as odds ratios (OR) with 95% confidence intervals (CI). We then used multivariate regression to adjust for variables associated with mortality in univariate analysis (p < 0.10). Each set of severity criteria was analysed in a separate multivariate model due to overlaps in criteria.

We assessed the performance of each set of severity criteria for predicting death in two ways. Firstly, we calculated the sensitivity, specificity, positive predictive value (PPV), and negative predictive value (NPV) for each set, expressed as percentages. For the severity scores that are not binary, we tested different cut-off values for classification as severe *versus* not severe and selected the best performing cut-off. Secondly, we produced receiver operator characteristics (ROC) curves for each set of criteria and calculated the area under the ROC curves (AUROC) with 95% CIs. The null hypothesis for ROC curve interpretation is that the AUROC = 0.5. An AUROC closer to 1.0 indicates better performance. Finally, we created novel severity criteria by adding clinical variables associated with mortality to high performing existing criteria, and calculated the AUC.

For all data management and analysis we used Microsoft Access 2007 and SPSS Statistics 17.0.

### Ethics and approvals

The study was approved by the Tayside Medical Research Ethics Committee A, who deemed that informed consent was not required as data were observational, extracted from routine care records, and anonymised for analysis. The Caldicott Guardian for inpatients gave permission to access the data.

## Results

### Mortality summary

There were 339 eligible patients in the first cohort and 302 in the second cohort. The two cohorts were the same in terms of age, gender, Charlson Index of comorbidity, and SIRS criteria (Chi-squared tests p > 0.2 for each comparison) so they were combined. Outcome data were missing for one patient, leaving 640 patients for analysis. Death within 30 days of sepsis onset occurred in 124/640 (19.4%, 95% CI 16.3-22.4%) patients. A further 56 patients died between 31 and 90 days, meaning 90 day mortality was 28.1% (180/640, 95% CI 24.6-31.6%). Mean survival among those who died within 90 days was 24.0 days (95% CI 20.8-27.2), and median survival 16.5 days (interquartile range (IQR) 7.0-36.0). Distribution of the cohort by demographic and clinical characteristics and six sets of severity criteria, along with the mortality in each variable category, are given in Table 2. The minimum number of SIRS criteria was two because of the sepsis case definition. Of note, in 24% (150/633) of cases blood cultures were positive, in 66% (418) they were negative and in the remaining 10% (65) a probable contaminant organism was isolated (Table 2 and Additional file 2: Table S2).

**Table 2:** Cohort distribution of mortality by demographic and clinical variables and by severity criteria

**Variable**	**Category**	**N in category**^**+**^	**30 day mortality**	**OR (95% CI) for death within 30 days**	**90 day mortality**	**OR (95% CI) for death within 90 days**
**Age group**	**<60 yrs**	171	8%	1.00	13%	1.00
	**60–69 yrs**	127	9%	1.06 (0.47–2.43)	10%	0.73 (0.36–1.51)
	**70–79 yrs**	174	20%	2.82 (1.46–5.47)**	31%	2.90 (1.68–4.99)**
	**≥80 yrs**	168	38%	6.90 (3.68–12.95)**	53%	7.43 (4.35–12.66)**
**Gender**	**Male**	351	18%	1.00	27%	1.00
	**Female**	289	21%	1.22 (0.83–1.81)	30%	1.16 (0.82–1.64)
**SIMD quintile**	**1 Deprived**	130	19%	1.00	26%	1.00
	**2**	107	21%	1.09 (0.57–2.06)	30%	1.21 (0.68–2.13)
	**3**	115	24%	1.35 (0.74–2.49)	36%	1.56 (0.91–2.70)
	**4**	161	18%	0.92 (0.51–1.67)	28%	1.10 (0.65–1.84)
	**5 Affluent**	111	18%	0.92 (0.48–1.77)	24%	0.91 (0.51–1.63)
**Charlson index**	**0**	133	12%	1.00	16%	1.00
	**1**	170	15%	1.33 (0.68–2.60)	24%	1.71 (0.95–3.06)
	**2**	137	22%	2.03 (1.05–3.93)*	32%	2.50 (1.39–4.49)**
	**≥3**	200	26%	2.57 (1.40–4.73)**	37%	3.13 (1.81–5.42)**
**Admission type**	**Elective**	129	3%	1.00	5%	1.00
	**Emergency**	511	23%	9.59 (3.47–26.50)**	34%	10.59 (4.57–24.51)**
**Ward type**	**Medicine**	281	30%	1.00	40%	1.00
	**Surgery**	244	11%	0.28 (0.17–0.45)**	16%	0.30 (0.20–0.45)**
	**Orthopaedics**	68	10%	0.27 (0.12–0.61)**	21%	0.39 (0.21–0.74)**
	**Other**	47	15%	0.41 (0.18–0.95)*	29%	0.64 (0.33–1.25)
**Length of stay prior to sepsis onset**	**1–3 days**	187	11%	1.00	17%	1.00
	**4–7 days**	139	18%	1.83 (0.97–3.45)	27%	1.83 (1.07–3.13)*
	**8–21 days**	213	23%	2.50 (1.42–4.38)**	33%	2.52 (1.56–4.06)**
	**>21 days**	101	29%	3.53 (1.88–6.63)**	40%	3.44 (1.98–5.98)**
**Blood culture result**	**Negative**	418	18%	1.00	26%	1.00
	**Contaminant**	65	22%	1.22 (0.64–2.31)	32%	1.39 (0.79–2.44)
	**Positive**	150	21%	1.20 (0.76–1.91)	34%	1.50 (1.00–2.24)*
**SIRS criteria**	**2**	227	13%	1.00	22%	1.00
	**3**	279	19%	1.60 (0.98–2.62)	29%	1.51 (1.01–2.27)*
	**4**	132	31%	3.08 (1.80–5.26)*	36%	2.08 (1.29–3.34)**
**SEWS score**	**0–1**	95	15%	1.00	26%	1.00
	**2–3**	242	15%	1.01 (0.52–1.97)	19%	0.66 (0.38–1.15)
	**4–5**	182	21%	1.53 (0.78–2.99)	35%	1.48 (0.86–2.57)
	**≥6**	121	30%	2.45 (1.23–4.88)**	38%	1.72 (0.96–3.09)
**CURB65 score**	**0**	112	3%	1.00	6%	1.00
	**1**	179	11%	4.57 (1.33–15.76)*	17%	3.14 (1.33–7.41)**
	**2**	190	23%	10.95 (3.31–36.19)**	34%	7.80 (3.43–17.74)**
	**≥3**	159	36%	20.30 (6.17–66.87)**	48%	14.09 (6.17–32.17)**
**IHI criteria**	**Not severe**	431	14%	1.00	23%	1.00
	**Severe**	207	29%	2.49 (1.66–3.72)**	39%	2.11 (1.48–3.02)**
**SSC criteria**	**Not severe**	468	14%	1.00	22%	1.00
	**Severe**	172	34%	3.24 (2.15–4.88)**	45%	2.87 (1.98–4.17)**
**Survive Sepsis criteria**	**Not severe**	450	15%	1.00	23%	1.00
	**Severe**	188	30%	2.53 (1.69–3.80)**	39%	2.13 (1.48–3.07)**

^+^Total N = 640 except for SIMD (N = 624) blood culture results (N = 633).

*p ≤ 0.05, **p < 0.01.

Odds ratios (OR) with 95% confidence intervals (CI) for likelihood of death associated with each variable category from univariate logistic regression are given.

### Logistic regression

In univariate logistic regression analysis, increasing age and comorbidity were associated with increased mortality. Having been admitted as an emergency, longer stays in hospital prior to sepsis onset, and being in a medical ward, were also each associated with increased mortality, but blood culture results had little association. Higher scores against all six sets of severity criteria were associated with increased mortality, but the SEWS score was least, and CURB65 most, discriminant (Table 2).

Gender and SIMD were excluded from multivariate analysis due to lack of association in univariate analysis. Other variables were included in the adjusted models for the severity criteria, except age was excluded from the CURB65 model because age forms part of the score. Older age remained associated with increased mortality after adjustment in all other models. Associations between comorbidity and death were much weaker, and only significant for 90 day mortality with Charlson Index ≥3 in some models (OR 1.73-2.13, p 0.03-0.09, across all models). Having been admitted as an emergency remained associated with increased 30 day mortality (OR 3.82-4.35, p ≤ 0.02 in all models). Being in a surgical, or orthopaedic, ward at sepsis onset was associated with lower adjusted 30 day mortality compared to being in a medical ward (OR 0.44-0.49, and 0.17-0.26, respectively across all models, p < 0.01 in all instances). Having an inpatient stay of >21 days before sepsis onset was also still associated with increased 30 day mortality (OR 2.81-3.10, p < 0.01 in all models) (data not shown). Blood culture results had no association with mortality in the adjusted models.

All sets of severity criteria were significantly associated with mortality in adjusted models (Table 3). For SIRS criteria and SEWS scores, associations with mortality were only significant in the highest category, and only at 30 days. Each increase in CURB65 score had a stepwise increase in the adjusted odds of death, with scores of three or more having 12 times increased risk of death compared to a score of zero (Table 3). The other three sets of severity criteria all had significant associations with mortality (Table 3), with similar odds ratios to those in the univariate models (Table 2).

**Table 3:** Adjusted likelihood of death associated with severity criteria category

**Severity criteria**	**Category**	**30 day mortality OR (95% CI)**	**P value**	**90 day mortality OR (95% CI)**	**P value**
**SIRS criteria**	**2**	1.00	-	1.00	-
	**3**	1.44 (0.83–2.49)	0.197	1.26 (0.78–2.03)	0.350
	**4**	**2.36 (1.29-4.33)**	**0.005**	1.39 (0.80–2.42)	0.242
**SEWS score**	**0–1**	1.00	-	1.00	-
	**2–3**	1.06 (0.50–2.24)	0.874	0.61 (0.32–1.17)	0.137
	**4–5**	1.36 (0.65–2.87)	0.413	1.36 (0.72–2.60)	0.346
	**≥6**	**2.20 (1.02–4.75)**	**0.045**	1.32 (0.66–2.63)	0.430
**CURB65 score**	**0**	1.00	-	1.00	-
	**1**	**3.90 (1.10–13.81)**	**0.035**	**2.48 (1.02–6.04)**	**0.046**
	**2**	**8.93 (2.60–30.67)**	**0.001**	**5.81 (2.45–13.75)**	**<0.001**
	**≥3**	**16.97 (4.92–58.55)**	**<0.001**	**10.39 (4.33–24.93)**	**<0.001**
**IHI criteria**	**Not severe**	1.00	-	1.00	-
	**Severe**	**2.64 (1.66–4.21)**	**<0.001**	**2.20 (1.42–3.40)**	**<0.001**
**SSC criteria**	**Not severe**	1.00	-	1.00	-
	**Severe**	**3.16 (1.98–5.05)**	**<0.001**	**2.68 (1.73–4.15)**	**<0.001**
**Survive Sepsis criteria**	**Not severe**	1.00	-	1.00	-
	**Severe**	**2.66 (1.65–4.27)**	**<0.001**	**2.12 (1.36–3.30)**	**0.001**

Odds ratios from multivariate logistic regression models. All models were adjusted for Charlson Index of comorbidity, admission type, ward type at sepsis onset, length of hospital stay prior to sepsis onset and blood culture result, and all models except CURB65 were adjusted for age.

### Performance characteristics of severity criteria

The performance characteristics for predicting 30 day mortality for each set of severity criteria are given in Table 4, including the mortality among those below the best cut-off value for each. SIRS criteria (cut-off of ≥3) had reasonable sensitivity, but had the lowest specificity of all criteria (Table 4). The SEWS score (cut-off ≥4) was more specific but at the expense of sensitivity. The CURB65 score (cut-off ≥2) performed best, with PPV almost as good as any other set of criteria and much stronger other performance characteristics (Table 4). CURB65 had the lowest mortality among patients below the cut-off, and the biggest difference between those above and below it (29% *versus* 8%). The other three sets of criteria had very similar performance characteristics, due to similar inclusion criteria (Table 4).

**Table 4:** Performance characteristics of each severity criteria

**Severity criteria**	**Performance characteristics for 30 day mortality**					**AUROC (95% CI)**	
	**Mortality below cut-off value**	**Sensitivity**	**Specificity**	**PPV**	**NPV**	**30 day mortality**	**90 day mortality**
**SIRS criteria**	13%	76%	38%	23%	87%	0.61	0.57
	(29/227)	(94/123)	(198/515)	(94/411)	(198/227)	(0.55–0.67)	(0.52–0.62)^b^
**SEWS score**	15%	60%	56%	24%	85%	0.60	0.59
	(50/337)	(74/124)	(287/516)	(74/303)	(287/337)	(0.54–0.66)	(0.54–0.65)
**CURB65 score**	8%	81%	52%	29%	92%	0.72	0.72
	(23/291)	(101/124)	(268/516)	(101/349)	(268/291)	(0.67–0.77)	(0.67–0.76)
**IHI criteria**	14%	50%	72%	29%	86%	0.61	0.59
	(62/431)	(61/123)	(369/515)	(61/207)	(369/431)	(0.55–0.66)	(0.54–0.64)
**SSC criteria**	14%	48%	78%	34%	86%	0.63	0.61
	(65/468)	(59/124)	(403/516)	(59/172)	(403/468)	(0.57–0.69)	(0.56–0.66)
**Survive Sepsis criteria**	15%	46%	75%	30%	85%	0.61	0.58
	(66/450)	(57/123)	(384/515)	(77/188)	(384/450)	(0.55–0.66)	(0.53–0.63)

^a^Positive predictive value equals the mortality rate above the cut-off value for each set of criteria.

^b^p = 0.004, (p ≤ 0.001 for all other AUROC).

Area under receiver operating characteristics curves AUROC = (with 95% CI) for the ability of each set of severity criteria to predict 30 and 90 day mortality.

**Figure 1:**
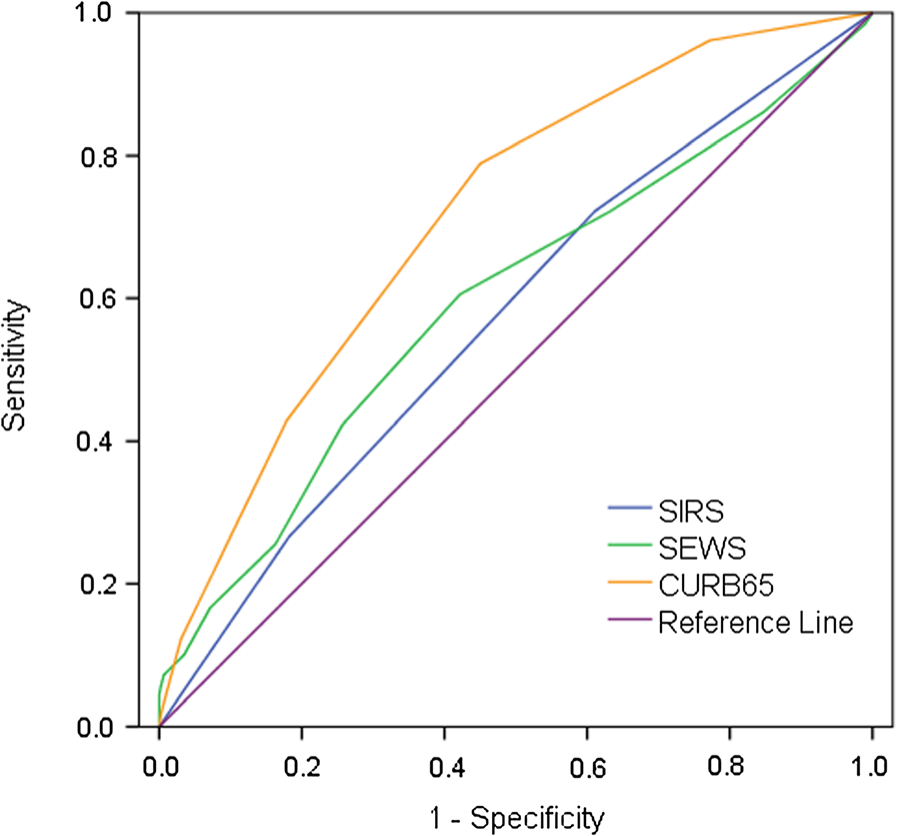
Area under receiver operating characteristics curves (AUROC) for 30 day mortality by SIRS criteria, SEWS score and CURRB65 score.

**Figure 2:**
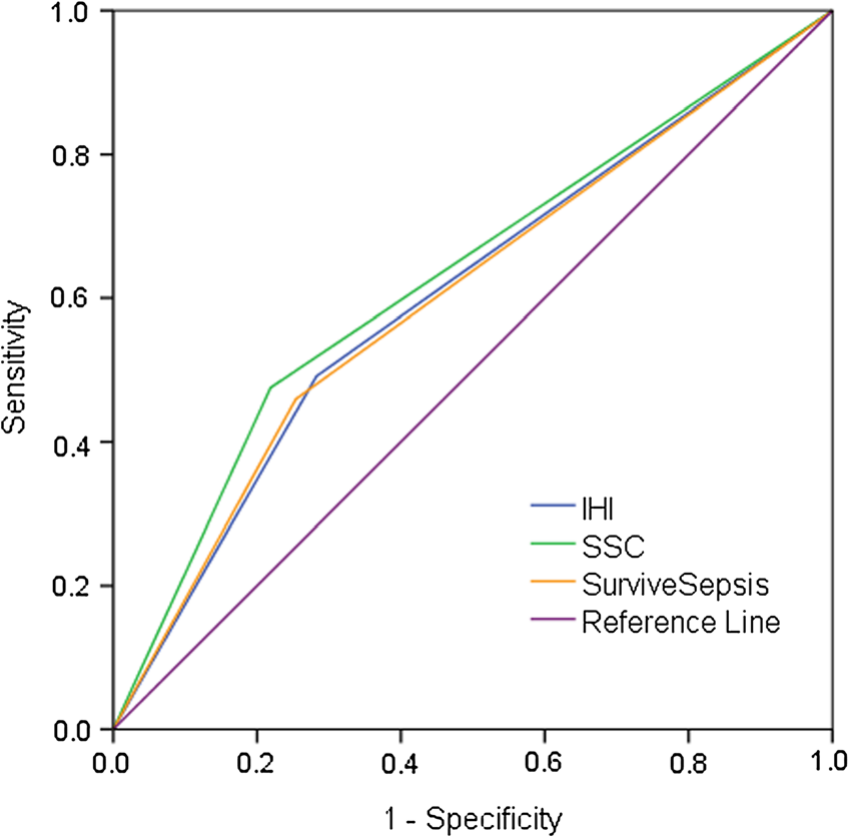
Area under receiver operating characteristics curves (AUROC) for 30 day mortality by IHI, SSC and Survive Sepsis severity criteria.

The AUROC with 95% CIs for each set of severity criteria, for 30 and 90 day mortality, are given in Table 4. The ROC curves for 30 day mortality are given in Figure 1 and Figure 2. CURB65 had the largest AUROC at 0.72 for both 30 and 90 day mortality. The other severity criteria had AUROC between 0.60 and 0.63 at 30 days, and 0.57 and 0.61 at 90 days (Table 4). Of the existing criteria, the CURB65 score performed best so we used it to test whether performance improved further with the addition of clinical variables that were significantly associated with mortality in the adjusted regression analysis. Thus, we allocated a point for each of: emergency admission; admitted ≥21 days prior to sepsis onset, and; medical ward at sepsis onset. Adding each of these points to the CURB65 score in different combinations resulted in small increases in AUROC (detailed in Additional file 3: Table S3). The addition of all three resulted in an AUC of 0.77 for both 30 and 90 day mortality (see Additional file 3: Table S3).

## Discussion

Among a large cohort of prospectively identified hospital inpatients who developed sepsis during their admission, we found high mortality rates that were highest among older patients, emergency admissions, patients in medical wards at sepsis onset, patients with longer inpatient stays prior to sepsis onset, and those who scored highly on any of the severity criteria tested. The CURB65 severity score for community-acquired pneumonia performed best at predicting mortality.

The 30 day mortality rate in this cohort was similar to that previously reported for septic patient populations [1,2,6,14]. Evidence is accumulating that the effect of sepsis on mortality extends beyond 30 days [21,22], and extending follow up to 90 days in our study detected 50% more deaths than at 30 days. Associations between mortality and ward type, and whether the admission was emergency or elective, suggest that these variables indicate comorbidity and/or frailty in addition to that detected by the Charlson Index. The Scottish standardised mortality ratio [23], used for national recording and reporting, accounts for these factors, indicating that such associations are established. The lack of associations between Charlson comorbidity and mortality in adjusted analysis may be partly because the Index was developed and validated for predicting mortality at one year among general inpatient populations [19], rather than at shorter timeframes among septic inpatients. The Charlson Index was developed in the 1980′s so is outdated, with excessive weighting for a diagnosis of “AIDS”, but it is still the most extensively validated comorbidity measure available. The study cohort only included one HIV positive patient so this limitation of Charlson will not have impacted significantly on the study results.

We found that blood culture results were not associated with mortality, consistent with others’ findings [14,24,25]. Obtaining positive blood cultures depends on many variables including the pathogen, host, site of infection, prior antibiotics, and sampling [26], so blood culture positivity is an inconsistent feature in sepsis of any severity. Sepsis studies only recruiting patients with positive blood cultures will miss many cases.

The SIRS criteria were designed to indicate the presence of the systemic inflammatory response syndrome, and not to predict outcome or for use as a graded severity scale. However, associations between the number of SIRS criteria and mortality in unselected blood culture patients [14] and patients with community-acquired pneumonia [15] have been observed. The SEWS score was designed to identify patients at risk of deterioration and cardiac arrest to aid prioritisation and organisation of urgent medical care, rather than to predict outcome. In a prospective audit of 435 patients, SEWS scores on admission correlated with in-hospital mortality (p < 0.001) and length of stay (p < 0.001), unadjusted for other variables [12]. SIRS and SEWS scores at sepsis onset did not perform as well in our adjusted analysis.

The CURB65 score was designed to risk-stratify patients with community-acquired pneumonia (CAP) on admission to hospital according to risk of death. It has been validated in large international cohorts in that context [16] but it has not been validated for use during the hospital admission [27]. The components of the CURB65 score (Table 1) are measures of organ dysfunction which in the context of sepsis indicate severe sepsis. This may explain why it performed well at predicting mortality in this cohort. A large prospective study of emergency department patients with any suspected infection reported that CURB65 scores correlated with in-hospital 28-day mortality, with increased odds of death of 2.4 (95% CI 2.0-3.0) with each score increase [17]. A cohort study comparing the performance characteristics of SIRS criteria, SEWS scores, and CURB65 scores in predicting mortality after CAP found CURB65 performed best [15]. The authors therefore argued against suggestions that generic sepsis severity scores could be applied to CAP patients [15,27]. Our findings indicate that the converse may be true, that severity scores developed for CAP can be applied to patients with sepsis from any source, at least those with onset in hospital.

The other three sets of published sepsis severity criteria come from clinical guidelines to aid the identification of patients with severe sepsis that may benefit from more aggressive therapy. The criteria and the number of patients identified as severe varied slightly but the performance characteristics were similar for all three, and were outperformed by CURB65 scores.

Two additional sets of criteria for risk stratification to predict mortality among blood culture patients [28], and deterioration among patients with early sepsis [29] have been proposed. Both are cumbersome, requiring multiple data items from a variety of sources and stratification into four levels of risk, and at least one has not been tested at individual patient level at the bedside [30]. The clinical application of severity tools has been described as dependent on three factors: 1) the accurate prediction of the outcome of interest; 2) the ability to classify patients into clinically useful groups (e.g. by level of risk); 3) simplicity [27,31]. The CURB65 score conforms to these factors, it is already in routine clinical use for CAP, four of the five required criteria are assessable clinically and the fifth (blood urea) a simple laboratory test available even in low technology settings. The addition of further clinical variables to the CURB65 score in our study led to only very small improvements in performance, the benefit of which would be outweighed by the added complexity and the potential difficulty in rapidly determining the additional variables. Biomarkers have been increasingly proposed as useful tools in identifying patients with infection and guiding therapy. However, biomarkers such as procalcitonin are not yet widely available and are expensive, and the added value of using procalcitonin across inpatient populations has not yet been demonstrated. One study found that a commonly measured and widely available inflammatory protein, C-reactive protein (CRP), improved the CURB65 AUROC for 30 day mortality among CAP patients [32], but this requires validation in further studies [27].

A major strength of this study was the combination of prospectively collected detailed clinical data with routinely collated data from population databases. There were very few missing data items, we used validated measures of comorbidity and deprivation, and we were able to follow patients up after hospital discharge. One limitation of applying severe sepsis criteria to this cohort was that blood lactate level in particular was not available for all patients. Another limitation in testing severity criteria, common to all such studies, is that the analysis is based on a one-off set of clinical recordings rather serial sets of recordings. In a prospective study of patients admitted with skin and soft tissue infections, we found that the worst set of recordings in the initial 24 hours was more predictive of illness severity and outcome than the first set [33], although prompt treatment relies on decision-making on the data available at onset.

## Conclusions

Severe sepsis, as identified by any severity criteria, has significant associated mortality. It is necessary to promptly identify high risk patients to aid therapeutic decision making, and guide prognosis. In selecting severity criteria there will always be a trade-off between high sensitivity (with the risk of over-treating some patients) and high positive predictive value (with the risk of missing some patients). We should also emphasise that any severity scoring system should be used along with, and not in place of, clinical judgement in therapeutic decision making. In our large cohort of septic hospital inpatients, the CURB65 score outperformed other measures of illness severity in predicting mortality, showing promise for such use in clinical practice. Testing in a validation cohort of septic patients, including those on admission to hospital, would help clarify its applicability.

## Abbreviations

AIDS: Acquired immune deficiency syndrome; AUROC: Area under receiver operator characteristics (curve); CAP: Community-acquired pneumonia; CHI: Community Health Index; CURB65: Confusion, urea, respiratory rate, blood pressure, age ≥ 65 years; HIC: Health Informatics Centre; HIV: Human immunodeficiency virus; IHI: Institute for Healthcare Improvement; NPV: Negative predictive value; PPV: Positive predictive value; ROC: Receiver operator characteristics (curve); SEWS: Standardised early warning system; SIMD: Scottish Index of Multiple Deprivation; SIRS: Systemic inflammatory response syndrome; SMR: Scottish Morbidity Record; SSC: Surviving Sepsis Campaign.

## Competing interests

All authors have declared that they have no competing interest.

## Author contributions

CM, BG, JE and PD conceived and designed the study and secured the funding. CM and JP collected the clinical data. CM analysed the data and wrote the first version of the manuscript. All authors critically reviewed the manuscript and approved the final version.

## Pre-publication history

The pre-publication history for this paper can be accessed here:

http://www.biomedcentral.com/1471-2253/14/1/prepub

## Supplementary Material

Additional file 1: Table S1 SEWS score detail.pdf contains a table entitled “Standardised early warning system (SEWS) scores allocated for each clinical observation” giving additional information on this scoring system with the relevant reference from the literature. Click here for file

Additional file 2: Table S2 Classification of blood culture isolates.pdf contains a table entitled “Classification of all organisms isolated from blood cultures in study patients” in which isolated organisms are classified into whether they are likely pathogens or contaminants. Click here for file

Additional file 3: Table S3 AUROC curves with extra variables.pdf contains a table entitled “AUROC curves for severity criteria created by adding further clinical variables to the CURB65 score” demonstrating changes made to the area under receiver operating characteristics curves made by adding clinical variables to the CURB65 score for community acquired pneumonia. Click here for file
