# Supplementary material for: Identifying which septic patients have increased mortality risk using severity scores: a cohort study
Source: BMC Anesthesiol. 2014 Jan 2;14:1. doi: 10.1186/1471-2253-14-1 (PMC3918178; doi:10.1186/1471-2253-14-1)
Supplement: Additional file 1: Table S1 — SEWS score detail.pdf contains a table entitled “Standardised early warning system (SEWS) scores allocated for each clinical observation” giving additional information on this scoring system with the relevant reference from the literature. [file 1471-2253-14-1-S1.pdf]

Standardised early warning system (SEWS) scores allocated for each clinical observation (adapted from [1]).

Parameter	Score						
	3	2	1	0	1	2	3
Respiratory rate (breaths/min)	≤8			9-20	21-30	31-35	≥36
Oxygen saturation (%)	<85	85-89	90-92	≥93			
Temperature (°C)	<34	34-34.9	35-35.9	36-37.9	38-38.4	≥38.5	
Systolic blood pressure (mmHg)	≤69	70-79	80-99	100-199		≥200	
Heart rate (beats/min)	≤29	30-39	40-49	50-99	100-109	110-129	≥130
AVPU response (stimulus required to induce response)	Unresp-onsive	Pain	Verbal	Alert			

Case example; a patient has respiratory rate = 26, oxygen saturation = 94%, temperature = 38.4, blood pressure = 84/62, heart rate = 107, and is alert, therefore SEWS = 4. The action required in response to the SEWS score is detailed on the charts and ranges from “Continue routine observation” with SEWS = 0, to “Call appropriate Registrar for immediate review” with SEWS ≥ 6. SEWS = 4 mandates immediate involvement of the nurse in charge and medical assessment within 20 minutes.

1. Paterson R, MacLeod DC, Thetford D, Beattie A, Graham C, Lam S, Bell D: **Prediction of in-hospital mortality and length of stay using an early warning scoring system: clinical audit.** *Clin Med* 2006, **6**(3):281-284.
