# Supplementary material for: Identifying which septic patients have increased mortality risk using severity scores: a cohort study
Source: BMC Anesthesiol. 2014 Jan 2;14:1. doi: 10.1186/1471-2253-14-1 (PMC3918178; doi:10.1186/1471-2253-14-1)
Supplement: Additional file 2: Table S2 — Classification of blood culture isolates.pdf contains a table entitled “Classification of all organisms isolated from blood cultures in study patients” in which isolated organisms are classified into whether they are likely pathogens or contaminants. [file 1471-2253-14-1-S2.pdf]

Classification of all organisms isolated from blood cultures in study patients

**Positive blood culture**	**Probable contaminant**
Acinetobacter lwoffi	Bacillus sp.
Actinobaculum urinale	Coagulase negative staphylococci
Anaerobic Gram positive cocci	Corynebacterium sp.
Anaerobic streptococci	Diphtheroids
Bacteroides fragilis	Gram positive bacilli
Candida	Micrococcus sp.
Candida albicans	Propionibacterium sp.
Candida glabrata	Staphylococcus capitis
Candida guillermondii	Staphylococcus epidermidis
Clostridium sp.	Staphylococcus haemolyticus
Enterococcus faecalis	Staphylococcus hominis
Enterococcus faecium	Staphylococcus warneri
Escherichia coli	
Group B streptococci	
Klebsiella oxytoca	
Klebsiella pneumoniae	
Pantoea sp.	
Proteus mirabilis	
Proteus vulgaris	
Pseudomonas aeruginosa	
Rhodotorula mucilaginosa	
Serratia liquefaciens	
Serratia marcescens	
Staphylococcus aureus	
Streptococcus anginosus	
Streptococcus gallolyticus	
Streptococcus milleri group	
Streptococcus mitis	
Streptococcus pneumoniae	
Streptococcus pyogenes	
