# Supplementary material for: Identifying which septic patients have increased mortality risk using severity scores: a cohort study
Source: BMC Anesthesiol. 2014 Jan 2;14:1. doi: 10.1186/1471-2253-14-1 (PMC3918178; doi:10.1186/1471-2253-14-1)
Supplement: Additional file 3: Table S3 — AUROC curves with extra variables.pdf contains a table entitled “AUROC curves for severity criteria created by adding further clinical variables to the CURB65 score” demonstrating changes made to the area under receiver operating characteristics curves made by adding clinical variables to the CURB65 score for community acquired pneumonia. [file 1471-2253-14-1-S3.pdf]

AUROC curves for severity criteria created by adding further clinical variables to the CURB65 score

**Proposed severity criteria**	**Area under ROC curve (95% confidence intervals)**	
	**30 day mortality**	**90 day mortality**
**CURB65 + medical ward (W)**	0.75 (0.70-0.80)	0.74 (0.70-0.78)
**CURB65 + emergency admission (A)**	0.75 (0.70-0.79)	0.75 (0.71-0.79)
**CURB65 + prior length of stay ≥21 days (L)**	0.73 (0.68-0.77)	0.72 (0.68-0.77)
**CURB65 + W + A**	0.77 (0.72-0.81)	0.76 (0.72-0.80)
**CURB65 + W + L**	0.76 (0.71-0.80)	0.75 (0.71-0.79)
**CURB65 + A + L**	0.75 (0.71-0.80)	0.75 (0.71-0.79)
**CURB65 + W + A + L**	0.77 (0.73-0.82)	0.77 (0.73-0.81)

$p<0.001$ in all instances
